# Residues of Pesticides and Heavy Metals in Polish Varietal Honey

**DOI:** 10.3390/foods11152362

**Published:** 2022-08-06

**Authors:** Monika Kędzierska-Matysek, Anna Teter, Piotr Skałecki, Barbara Topyła, Piotr Domaradzki, Ewa Poleszak, Mariusz Florek

**Affiliations:** 1 Department of Quality Assessment and Processing of Animal Products, University of Life Sciences in Lublin, Akademicka 13, 20-950 Lublin, Poland; 2 Chair and Department of Applied and Social Pharmacy, Laboratory of Preclinical Testing, Medical University of Lublin, Chodźki Street 1, 20-093 Lublin, Poland

**Keywords:** honey, pesticide residues, QuEchERS, fungicide, insecticide, veterinary drugs

## Abstract

The levels of chemical pollutants were determined in 30 samples of varietal honey from southeastern Poland, including 223 pesticides (insecticides, herbicides, fungicides, acaricides, plant growth regulators, and veterinary drugs) and 5 heavy metals (Pb, Cd, Hg, Cu, and Zn). In 10% of the samples, no pesticide residues were found. The most frequently identified pesticides were thiacloprid (90% of the samples, max 0.337 mg/kg), acetamiprid (86.6%, max 0.061 mg/kg), carbendazim (60%, max 0.049 mg/kg), DMF (56.6%, max 0.038 mg/kg), total amitraz (53.3%, max 0.075 mg/kg), thiamethoxam (26.6%, max 0.004 mg/kg), thiacloprid-amide (13.3%, max 0.012 mg/kg), dimethoate (10%, max 0.003 mg/kg), azoxystrobin (10%, max 0.002 mg/kg), tebuconazole (6.66%, max 0.002 mg/kg), and boscalid (3.33%, max 0.001 mg/kg). The acceptable limits for the compounds were not exceeded in any sample. The Pb content ranged between 0.044 and 0.081 mg/kg. The concentration of Hg and Cd did not exceed 5.0 µg/kg and 0.02 mg/kg, respectively. The honey variety significantly (*p* < 0.01) influenced the content of Cu, which ranged from 0.504 (rapeseed honey) to 1.201 mg/kg (buckwheat). A similar tendency (*p* > 0.05) was observed for the Zn content, which ranged from 0.657 mg/kg (linden) to 2.694 mg/kg (buckwheat). Honey produced in southeastern Poland was shown to be safe for human consumption.

## 1. Introduction

Honey, due to its high nutritional value and numerous health-promoting properties, is a valuable component of the daily diet. The chemical composition of honey is varied and depends mainly on the region where it is produced, the soil conditions, and the water and air quality, which affect the quality of the food sources for bees, including the presence and level of chemical pollutants [1]. The health risks associated with honey consumption stem from the presence of pesticide residues (including active substances and their metabolites), antibacterial substances (including antibiotics), and heavy metals. Another source of risk in honey is microbial contaminants [2]. These substances come mainly from the environment, whose cleanliness depends largely on human activity. Antibacterial substances are used to prevent and fight diseases in broods and bees, and pesticides are meant to protect crop plants against fungi (fungicides), insects (insecticides), and weeds (herbicides). The use of pesticides in agriculture is essential to obtaining high yields, but results in the contamination of the soil, water, air, and also the flowers from which bees collect nectar and pollen, the natural components of honey [3]. These agents negatively affect both bees and people, causing changes in the endocrine and nervous systems [4]. Detailed information on the potential sources of the contamination of honeys (e.g., heavy metals, airborne particulate matter, and agrochemical pesticides) was provided by Cunningham et al. [5]. Therefore, the monitoring of their levels in food products is crucial for ensuring consumer health safety. 

Colony collapse disorder (CCD) threatens the health of beehives worldwide, but scientists still struggle to identify the specific causes. In view of the important ecological and economic value of bees, there is a need to monitor and maintain healthy bee stocks. In the framework of the Farm to Fork Strategy, one of the main priorities of the European Commission is the 50% reduction of the overall use of (and risk from) chemical pesticides by 2030, especially for the most hazardous ones. In addition, the EU Pollinators Initiative objectives state that by 2030, the scientific knowledge about the magnitude, causes, and consequences of the insect pollinator decline will have improved, that the main known causes of this decline will be addressed and managed, and that the societal awareness and collaboration amongst stakeholders will have strengthened [6]. In response to a mandate from the European Parliament’s Committee for the Environment, Public Health, and Food Safety (ENVI), The European Food Safety Authority (EFSA) devised an integrated framework for the environmental risk assessment (ERA) of multiple stressors in honey bees (MUST-B). These stressors range from chemicals such as plant protection products, other types of chemicals (e.g., biocides), biological agents (e.g., *Varroa*, *Nosema*), and other elements (e.g., food availability, weather conditions, and beekeeping management practices) in managed honey bees [7].

Among the compounds recognized as toxic for pollinating insects, an important group is the neonicotinoid insecticides, widely used in agriculture and with a share of about one third of the global insecticide market. Neonicotinoids act on nicotinic acetylcholine receptors (nAChRs) in the central nervous system of the honeybee and other pollinating insects, which impairs their learning and memory functions, causing them to not look for food [8] and thus leading to their elimination [9]. The most commonly used insecticides are imidacloprid and acetamiprid [10].

The Agency for Toxic Substances and Disease Registry [11] lists polychlorinated biphenyls, dimethoate, and metals such as lead, mercury, cadmium, zinc, and copper as harmful substances. Fakhri et al. [12], based on a meta-analysis of the results of 45 studies, estimated the overall rank order of nine potentially toxic elements (PTE) according to their concentrations in honey (Fe > Mn > Pb > Cr > Cu > Ni > Cd > As > Hg) as well as their rank according to their hazard quotient (HQ: Pb > Cd > Mn > Fe >Ni > As > Cu > Hg > Cr). Lead (Pb) and cadmium (Cd), due to their carcinogenic and cytotoxic properties, are regarded as the most toxic heavy metals. Lead, mainly from car exhaust, is not transported by plants, but can pollute the air and subsequently nectar and honeydew. Cadmium from the metallurgical industry and combustion plants is transferred from the soil to plants; thus, it can contaminate nectar and honeydew [13]. The predominant source of lead, cadmium, mercury, and arsenic is industrial contamination, i.e., exhaust gases and fumes, as well as pesticides and synthetic fertilizers. Oroian et al. [14] showed that information on the level of heavy metals in honey can be used to determine its botanical origin, with an about 81% accuracy, and its geographic origin, but with only a 21% accuracy. 

One of the effects of human activity on the environment may be the presence of unacceptable chemical residues and drugs in the honey made by bees. The presence of these compounds is a significant challenge in monitoring the quality of honey. Taking into account human exposure to the effects of active substances and their potential cumulative and synergistic effects, maximum residue levels (MRL) have been established. Foodstuffs, including honey, are safe for human health or life if their content of these compounds does not exceed the acceptable limits [15]. The maximum residue levels of pesticides in food are regulated by Regulation (EC) No 396/2005 of the European Parliament and of the Council [16], and the levels of veterinary agents by Regulation (EC) No 37/2010 [17]. In Poland, honey is monitored in accordance with the Regulation of the Minister of Agriculture and Rural Development [18]. Specifically, it is tested for the presence of antibacterial substances, including sulphonamides and quinolones; medicinal products (carbamates and pyrethroids); chemical pollutants such as organochlorine pesticides, polychlorinated biphenyls (PCB), and organophosphate pesticides; and toxic elements. Changing hazards, however, make it necessary to study new unsafe chemical compounds as they appear. 

Testing for the presence of chemical contaminants in honey can provide important data on the presence of these contaminants in the environment. The honey bee (*Apis mellifera* L.) and its products are currently also used as bioindicators of environmental contamination. These insects fly around nectar plants growing up to 4 km away from the hive, but they can cover distances even up to 12 km, accumulating pollutants present in the air, soil, and water [19]. For this reason, honey can serve as an indicator material for evaluating the contamination of the environment from which bees have collected nectar for making honey. 

The aim of the study was to assess the safety of honey from southeastern Poland based on the levels of residues of pesticides, including organochlorine and organophosphate insecticides, herbicides, and fungicides; plant growth regulators; acaricides; and others, as well as the content of copper and zinc and the presence of these toxic heavy metals: lead, cadmium, and mercury. 

## 2. Materials and Methods

### 2.1. Material

The study was conducted on 30 samples of honey produced in 2019 from nectar and honeydew sources located in the Lublin region (southeastern Poland). The material comprised 10 samples of multifloral honey (MF), 6 samples of linden honey (LI), 5 samples of rapeseed honey (RS), 5 samples of buckwheat honey (BW), and 4 samples of honeydew honey (HD). Honey samples represented the locally produced honey from apiaries located in 8 districts from different parts of the Lubelskie voivodship: northern (Bialski district (BW *n* = 2, LI *n* = 1, RS *n* = 1, MF *n* = 1)), southern (Zamojski (MF *n* = 2, HD *n* = 1) and Biłgoraj (HD *n* = 1, LI *n* = 1, BW *n* = 2) districts), eastern (Włodawa (MF *n* = 2, LI *n* = 2, HD *n* = 1) and Chełmski (MF *n* = 2, RS *n* = 1) districts), western (Puławy (LI *n* = 2, HD *n* = 1) and Opole (MF *n* = 2, RS *n* = 1, BW *n* = 1) districts), and central (Lubelski district RS *n* = 2, MF *n* = 1). The apiaries were located in agricultural farmlands without concentrations of industry. The honey was purchased directly from beekeepers just after harvesting (from May to August) and stored in glass jars at 20 °C (±2 °C) out of direct sunlight. 

### 2.2. Determination of Pesticide Residues

Pesticide residues in the honey were determined by the QuEChERS (quick, easy, cheap, effective, rugged, and safe) method using chromatographs coupled with tandem mass spectrometers (LC-MS/MS and GC-MS/MS) [20]. The honey was tested for the presence of 223 substances: 93 insecticides, 57 herbicides, 57 fungicides, 9 acaricides, 4 veterinary drugs, and 3 plant growth regulators (a detailed list of the compounds analysed is presented in Table 1).

#### 2.2.1. LC-MS/MS

The Agilent series 1260 HPLC system was used for the analyses. The substances were separated on a Luna 3 µm Phenyl-Hexyl 150 mm × 2.0 mm column (Phenomenex, Torrance, NJ, USA) using water with 5 mM ammonium formate and acetonitrile as the mobile phase. The flow rate was 400 µL/min and the column was thermostated at 50 °C. Gradient elution was used. The injection volume was 2 µL, and the total LC analysis time was 40 min. Spectrometric analysis was performed using the AB Sciex QTRAP^®^ 6500 LC-MS/MS system (Framingham, MA, USA) with the Turbo Spray ion drive with positive ionization and positive and negative ionization. The spray voltage was set to 5000 V and −4500 V for positive and negative ionization, respectively. The source temperature was set to 550 °C. Nitrogen was used as the curtain gas (20 psi), collision gas (medium), and ion source gases, nebulizer gas (50 psi) and heating gas (55 psi). Analyst 1.6.2 software (AB Sciex, Framingham, MA, USA) was used to control the LC-MS/MS system and to archive the data.

#### 2.2.2. GC-MS/MS

A GC-MS/MS system with an Agilent 7890A+ gas chromatograph (Palo Alto, CA, USA), 7693B autosampler, split/splitless injector, and 7000B tandem mass spectrometry detector with an electron ionization source was used for the analysis. Chromatographic separation was carried out on an HP-5 MS UI capillary column (30 m × 0.25 mm ID, 0.25 µm, Agilent Technologies, Palo Alto, CA, USA) using helium with 99.9999% purity as the carrier gas (constant flow 0.9 mL/min). The injection volume was 1 μL. The following furnace temperature program was used: initial temperature 80 °C held for 1 min, increased by 40 °C/min to 200 °C, 2.3 °C/min to 210 °C (held for 5 min), and increased by 10 °C/min to 320 °C. The analysis time was 38 min. The remaining conditions were as follows: inlet temperature was 280 °C, transfer line temperature was 295 °C, source temperature was 300 °C, MS1 and MS2 quadrupole temperatures were 150 °C, collision gas flow rate (N_2_) was 1.5 mL/min, and quenching gas flow rate (He) was 2.25 mL/min. Mass Hunter B.07.01 software was used to control the GC-MS/MS system and to archive the data.

### 2.3. Determination of Heavy Metals (Pb, Cd, Hg, Cu, and Zn)

The levels of Cd and Pb in the digest solution of honey were determined according to Kędzierska-Matysek et al. [21] by inductively coupled plasma mass spectrometry (Varian MS-820ICP Mass Spectrometer). The gas used to create the plasma was argon (Messer) with 99.999% purity. No reaction chamber (CRI) was used in the analysis. The following settings were used: plasma flow at 16 dm^3^/min, nebulizer flow at 0.98 dm^3^/min, RF power of 1.38 kW, and sampling depth of 6.5 mm. The following isotopes of the elements were used: ^114^Cd, ^206^Pb, ^207^Pb, and ^208^Pb. 

Levels of Cu and Zn were analysed with a Varian SpectrAA 240 FS atomic absorption flame spectrometer (Fast Sequential Atomic Absorption Spectrometer, Varian Australia Pty Ltd., Mulgrave, Australia). The following settings were used for Cu: absorption—324.8 nm, slit width—0.5 nm, lamp current—4 mA. The corresponding settings for Zn were 213.9 nm, 1.0 nm, and 5 mA. The atomizer was a slit burner 100 mm in length operating on a stoichiometric acetylene/air gas mixture. During the analysis of Pb, Cd, Cu, and Zn, quality control was carried out by measuring blank samples and the certified reference material NCS ZC 73014 Tea. The results were expressed as mg/kg fresh weight.

Mercury content was determined using an AMA 254 atomic absorption spectrometer. The analysis was performed without mineralization, which limited the risk of contamination of the sample. Before each measurement, the apparatus was cleaned with air and deionized water. 

### 2.4. Data Analysis

All statistical analyses were performed using Statistica ver. 13 (TIBCO Software Inc., Palo Alto, CA, USA). The parametric and nonparametric descriptive statistics are presented in the tables and figures. The influence of honey variety on concentration of metals was verified by the Kruskal–Wallis test (comparison of multiple independent groups). Statistical differences between means at confidence levels of 95% and 99% (*p* < 0.05 and *p* < 0.01, respectively) were considered significant.

## 3. Results and Discussion

### 3.1. Pesticide Residues

Among the 223 pesticides analysed in the honey samples (Table 1), 11 substances were identified, including 5 insecticides (acetamiprid, thiacloprid, thiacloprid-amide, thiamethoxam, and dimethoate), 4 fungicides (carbendazim, azoxystrobin, tebuconazole, and boscalid), and 2 pharmacologically active substances used in veterinary medicine (DMF and total amitraz) (Table 2). No residues of herbicides, plant growth regulators, or acaricides were found in the honey samples. Only three samples were free of pesticide residues, but it should be noted that there was no sample in which the acceptable level of any of the substances was exceeded.

#### 3.1.1. Insecticides

Thiacloprid was detected in 90% of honey samples (max 0.337 mg/kg), acetamiprid in 86.7% (max 0.061 mg/kg), carbendazim in 60% (max 0.049 mg/kg), DMF in 56.7% (max 0.038 mg/kg), amitraz in 53.3% (max for total 0.075 mg/kg), thiamethoxam in 26.7% (max 0.004 mg/kg), thiacloprid-amide in 13.3% (max 0.012 mg/kg), dimethoate in 10% (max 0.003 mg/kg), azoxystrobin in 10% (max 0.002 mg/kg), tebuconazole in 6.66% (max 0.002 mg/kg), and boscalid in 3.33% (max 0.001 mg/kg) (Table 3). All the samples of rapeseed honey contained residues of thiacloprid, acetamiprid, and carbendazim (0.0702 mg/kg, 0.0300 mg/kg and 0.0242 mg/kg), and all the samples of multifloral honey contained thiacloprid and acetamiprid (0.1062 mg/kg and 0.0150 mg/kg) (Table 4). The highest degree of contamination with pesticides was noted for multifloral honey (0.1646 mg/kg in total) and rapeseed honey (0.1498 mg/kg), while buckwheat (0.0324 mg/kg), honeydew (0.0125 mg/kg), and linden (0.0268 mg/kg) honey were less contaminated with pesticides. All the samples of buckwheat honey were contaminated with the neonicotinoid insecticide thiacloprid (0.0122 mg/kg). Mitchell et al. [19], in an analysis of the presence of five commonly used neonicotinoids (acetamiprid, clothianidin, imidacloprid, thiacloprid, and thiamethoxam) in 198 samples of honey from various parts of the world, showed the regional differences in the use of different types of pesticides. Imidacloprid was predominant in honey from Africa and South America, acetamiprid in samples from Asia, thiamethoxam in honey from Oceania and North America, and thiacloprid in European honey. Under the Commission Implementing Regulation (EU) No 2020/23 [23], the approval of thiacloprid as an active substance was not renewed, and stores of it were to be used by 3 February 2021. At the same time, the European Food Safety Authority (EFSA) indicated a problem raising serious concern associated with the contamination of groundwater by the metabolites of thiacloprid [24]. All the suggested applications of thiacloprid entail the risk of exceeding the acceptable limit (0.1 μg/L) of the metabolites M30, M34, and M46 in drinking water. These metabolites are assumed to have the same carcinogenic properties as the original active substance (thiacloprid), which—according to Regulation (EC) No 1272/2008 of the European Parliament and of the Council [25] (amended by Commission Regulation (EU) 2019/521 [26])—is a category 2 carcinogen. This category includes agents, mixtures, and groups of agents for which there is sufficient evidence of carcinogenicity in humans as well as those for which there is no evidence of carcinogenicity in humans, but where there is evidence of carcinogenicity in experimental animals. In addition, thiacloprid is classified as a category 1B reproductive toxin. This group includes compounds presumed to adversely affect reproduction in humans based on experiments in animals. 

The contamination of honey with neonicotinoid insecticides largely depends on the apiary’s location in an agricultural area transformed by human activity [9]. A comparison was made of 90 samples of honey from western France, obtained from an apiary located on a plain and surrounded by crops and from another apiary situated in a bocage environment (fields surrounded by shrubs and trees). The levels of thiacloprid and thiamethoxam were higher in the honey from the apiary in the plain (11.6 ng/g and 2 ng/g) than in the honey from the bocage (9.1 ng/g and not detected). On the other hand, the maximum level of acetamiprid in the honey from the bocage (112.8 ng/g) was higher than in the honey from the plain (51.9 ng/g). 

Our study found no residues of organochlorine insecticides in the honey samples. Wilczyńska and Przybyłowski [27], on the other hand, detected eight organochlorine insecticides in honey from Poland, including HCH and *p*,*p*′-DDT (about 60% of samples), *p*,*p*-methoxychlor (29% of samples), and aldrin (21% of samples). Organochlorine pesticides are especially hazardous in agriculture due to their persistence and bioaccumulation in the environment, and their residues are identified in honey in various parts of the world. 

Ruiz-Toledo et al. [28] demonstrated the presence of a wide spectrum of organochlorine compounds in honey from Mexico (in the state of Chiapas), despite the fact that their use has been banned there since 2000. At least one organochlorine pesticide was present in more than 90% of honey samples—the most frequently identified were heptaclor (44% of samples), γ-HCH (36%), DDT (19%), endrin (18%), and DDE (11%).

The EFSA [24] reported that among 1301 samples of honey and other bee products evaluated in 2019, 78.7% were free of pesticide residues, and 20.4% of samples contained residues at the maximum level (MRL) or lower. The MRL was exceeded in 0.9% of samples. In total, 27 pesticides were quantified: most frequently thiacloprid (173 samples), acetamiprid (49 samples), amitraz (37 samples), dimoxystrobin (29 samples), azoxystrobin (27 samples), glyphosate (17 samples), coumaphos (10 samples), and flonicamid (10 samples). The MRL was exceeded for amitraz (four samples), glyphosate (two samples), and in one sample each for acetamiprid, bromide ion, thiacloprid, azoxystrobin, boscalid, and chlorfluazuron. 

El-Nahhal [29] identified residues of 92 pesticides in honey from 27 countries, including 6 substances belonging to toxicity class IA (extremely hazardous), 8 from class IB (highly hazardous), 42 from class II (moderately hazardous), 35 from class III (slightly hazardous), and one from class IV (not posing a serious threat). The hazard indices (HI) indicated a high potential health risk from honey consumption. 

Bargańska et al. [30], in an analysis of residues of 30 pesticides in honey from northern Poland (Pomerania), detected them in 29% of samples. In five samples (11%), the MRL was exceeded for bifenthrin (14.5 ng/g), fenpyroximate (16.3 ng/g), methidathion (25.7 ng/g), spinosad (20.6 ng/g), thiamethoxam (20.2 ng/g), and triazophos (20.3 ng/g). The organophosphate pesticide profenofos, which was not found in the present study, was detected as well (from <LOQ to 17.2 ng/g). In turn, Gaweł et al. [20] monitored 155 samples of Polish honey for the potential presence of 207 pesticide residues from 2015–2017. A total of 21 pesticides were identified: thiacloprid, acetamiprid, carbendazim, DMF and DMPF (amitraz metabolites), azoxystrobin, tebuconazole, dimethoate, boscalid, coumaphos, cyproconazole, flutriafol, tau-fluvalinate, tetraconazole, diazinon, dimoxystrobin, *p*,*p*′-DDD, difenoconazole, lindane, propiconazole, and prothioconazole-desthio. The most frequently detected pesticides were two cyano-substituted neonicotinoids—thiacloprid and acetamiprid—and carbendazim, which were found in 68%, 55%, and 38% of honey samples, respectively. In the present study, the presence of residues of these substances was detected in 90%, 87%, and 60% of samples. Moreover, Gaweł et al. [20] reported acetamiprid concentrations in honey ranging from 0.001 to 0.13 mg/kg and thiacloprid concentrations from 0.001 to 0.2 mg/kg. The maximum content of acetamiprid in the present study (0.061 mg/kg) was half of that reported in the cited study, while that of thiacloprid (0.337 mg/kg) was higher. 

#### 3.1.2. Fungicides

In the present study, the most frequently identified fungicide was carbendazim (present in 60% of samples), followed by azoxystrobin (10%), tebuconazole (6.66%), and boscalid (3.33% samples). Similarly, Gaweł et al. [20] showed the presence of the fungicides carbendazim (38%), azoxystrobin (11%), tebuconazole (10%), and boscalid (5%) in Polish honey, as well as other fungicides that were not detected in our study, including cyproconazole (6%), flutriafol (5%), tetraconazole (3%), dimoxystrobin, difenoconazole, propiconazole, and prothioconazole-desthio (1% each).

Only two honey samples contained both a triazole fungicide (tebuconazole) and cyano-substituted neonicotinoids (acetamiprid and thiacloprid). This combination of pesticide residues increases the toxicity of cyano-substituted neonicotinoids [31]. The degradation of cyano-substituted neonicotinoids (acetamiprid and thiacloprid) takes longer than in the case of nitro-substituted neonicotinoids (thiamethoxam, clothianidin, and imidacloprid). 

#### 3.1.3. Residues of Veterinary Drugs

The veterinary drugs whose residues were identified in the present study were DMF (*N*-2,4-Dimethylphenyl-formamide) and amitraz (total). Amitraz is used in apiaries against parasitic mites *Varroa destructor*, which are carriers of pathogenic viruses such as Acute Bee Paralysis Virus (ABPV) and Deformed Wing Virus (DWV) [32]. O’Neal et al. [33], however, showed that Amitraz has certain limitations, because exposure to this compound can adversely affect bees’ resistance to viral infections. Amitraz and its metabolites significantly alter the heart rate of the honey bee, most likely through interactions with octopamine receptors. A sublethal dose of amitraz can affect the detoxification, cyclic adenosine monophosphate (cAMP)-dependent protein kinase, immunity, antioxidant capacity, and the development of honeybee queens [34]. Amitraz is not a highly stable substance; in addition to amitraz itself, products of its degradation may be found, such as DMPF (*N*-(2,4-dimethylphenyl)-*N*′-methylformamide) and DMF (2,4-dimethylformanilide) [35]. 

### 3.2. Concentration of Metals

Honey is a plant and animal product that contains macro- and microelements as well as heavy metals (Pb, Cd, Hg, Cu, and Zn). Their concentrations vary depending on regional and environmental conditions, seasonal differences, and beekeeping and agricultural techniques [36]. 

Mercury is considered to be the most toxic heavy metal in the environment [37]. In the present study, the LOQ for Hg (5.0 µg/kg) was not exceeded in the honey. Brodziak-Dopierała et al. [38] detected the presence of Hg in 32 honeys from different parts of Poland (on average 0.37 µg/kg). The highest Hg content was noted in honeydew honey (1.55 µg/kg) and the lowest in goldenrod honey (0.02 µg/kg). In honey from Romania, the average Hg level was 0.908 µg/kg, ranging from 0.369 µg/kg to 2.154 µg/kg [14].

The level of elements in honey is associated with pollution in the area in which bees fly around honey plants. According to EC Regulation 1881/2006 [39], honey (as a food product) must meet the requirements for the maximum level of Pb (0.10 mg/kg). However, no limit has been established for Cd [36]. Tomczyk et al. [40] tested levels of Cd and Pb in successive links of the food chain (soil-plant-bee-honey) and showed that bees are susceptible to their bioaccumulation but at the same time are a biological barrier preventing the transfer of these elements to honey. 

**Figure 1:**
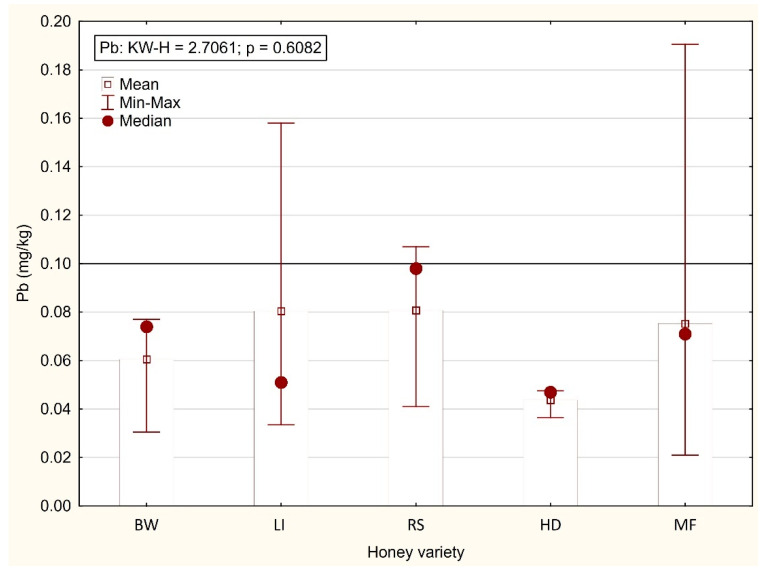
Content of Pb (mg/kg) in varietal honeys (BW—buckwheat; LI—linden; RS—rapeseed; HD—honeydew; MF—multifloral).

In the present study, the lowest Pb content was noted in honeydew honey (0.044 mg/kg) and the highest in linden (0.080 mg/kg) and rapeseed (0.081 mg/kg) honey (Figure 1). Due to the considerable variation in the Pb concentration in the honey samples, the differences were not confirmed statistically. The acceptable Pb concentration was exceeded in four honey samples, including one sample of rapeseed honey (0.107 mg/kg), two samples of linden honey (0.158 and 0.114 mg/kg), and one sample of multifloral honey (0.191 mg/kg). Piven et al. [2] reported a higher Pb content in Ukrainian honey (from the Odessa region), with the highest concentration noted in sunflower honey (0.24 mg/kg) and the lowest in multifloral honey (0.13 mg/kg). According to the authors, the high Pb level in honey was caused by the proximity of the apiaries to traffic routes. Aghamirlou et al. [41] reported a Pb content of 0.45 mg/kg in multifloral honey from Iran. An excessive Pb content in honey poses a threat to people mainly due to its neurotoxic and nephrotoxic effects [42]. 

The Cd content in the present study did not exceed the LOQ of 0.02 mg per kg of honey, except for one sample of linden honey (0.0215 mg/kg). Piven et al. [2] reported Cd at a level of 0.03 mg/kg in sunflower honey from the Odessa region (Ukraine). Aghamirlou et al. [41] detected Cd at a level of 0.013 mg/kg in multifloral honey from Iran. 

**Figure 2:**
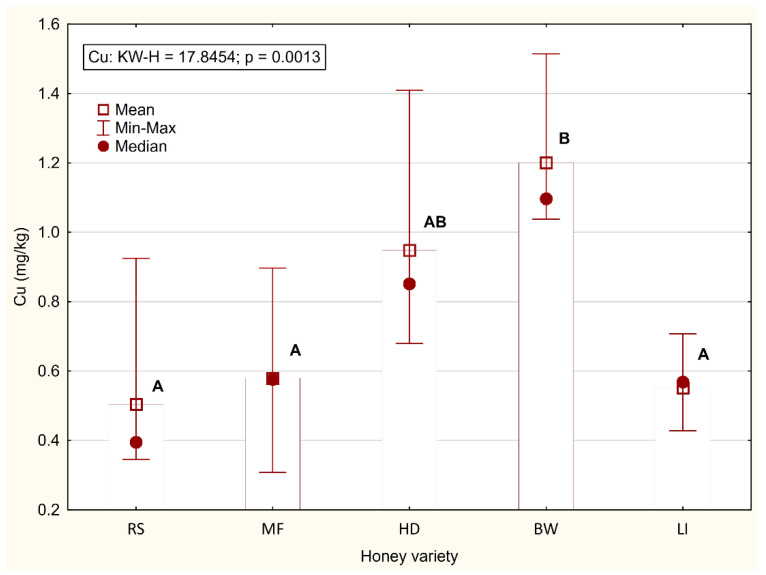
Content of Cu (mg/kg) in varietal honeys (RS–rapeseed; MF–multifloral; HD—honeydew; BW—buckwheat; LI—linden). Means with different letters (A, B) differ significantly (*p* < 0.01).

The Cu content in the honey was significantly influenced by the variety (*p* = 0.0013) (Figure 2). The Cu content was significantly the highest in buckwheat honey (1.201 mg/kg) and the lowest in rapeseed, linden, and multifloral honey (0.504–0.579 mg/kg). Dżugan et al. [43], in an analysis of Polish varietal honeys from the Podkarpacie region (southeastern Poland), showed a lower average Cu content in multifloral (0.21 mg/kg), rapeseed (0.05 mg/kg), linden (0.20 mg/kg), and honeydew (0.03 mg/kg) honey, and the highest Cu content in buckwheat honey (0.86 mg/kg), as in the present study. Tarapatskyy et al. [44], in honey obtained in Pogórze Karpackie (southern Poland), reported a Cu content similar to the levels found in the present study in multifloral (0.410 mg/kg), linden (0.460 mg/kg), and honeydew (0.960 mg/kg) honey.

**Figure 3:**
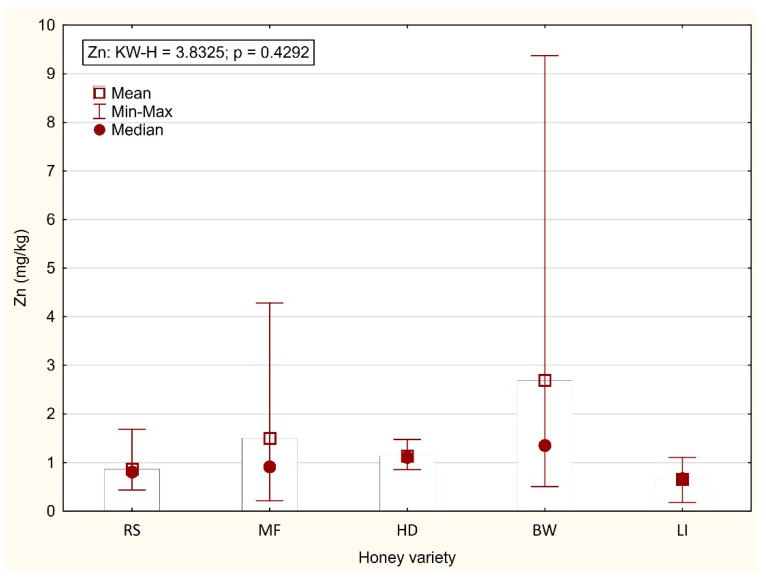
Content of Zn (mg/kg) in varietal honeys (RS—rapeseed; MF—multifloral; HD—honeydew; BW—buckwheat; LI—linden).

The honey variety did not affect the Zn level, although it was highest in buckwheat honey (2.694 mg/kg) compared with the other varieties (0.657–1.500 mg/kg) (Figure 3). Dżugan et al. [43] reported similar results for Zn in honey varieties, with the exception of honeydew honey, in which the level was twice as high as in our study (2.33 mg/kg). Similarly, in the study by Tarapatskyy et al. [44], the Zn level in honeydew honey from Pogórze Karpackie (Poland) was twice as high as in the present study, while its content in buckwheat honey was only a third as high. In general, the literature reports indicate a considerable variation in the content of the elements analysed (macro- and microelements and toxic metals) in European varietal honeys from Poland [21,45,46], Slovakia [44,47], Romania [14], and Turkey [36,48]. The contamination of honey with toxic metals is mainly associated with industry and agriculture [45]. Numerous studies supply valuable information on the effect of the environment on the quality of honey produced in industrial areas. Honey obtained from industrialized areas or near major roads has been shown to have higher concentrations of heavy metals.

Bartha et al. [49] analysed the content of heavy metals (Pb, Cd, Zn, and Cu) in multifloral honey produced in an industrial area of Romania, considered to be one of the most polluted regions of Eastern Europe (the town of Copșa Mică and its vicinity). The spread of pollutants emitted by the local industrial platform resulted from the topography of the area, where the main air masses are directed towards the corridors of the rivers Târnava Mare and Visa. Apiaries situated in the valley channeling pollutants from the industrial platform were shown to be more susceptible to the bioaccumulation of Pb than those situated in side valleys further from the source of pollution. The Cd concentration in the honey decreased exponentially with an increasing distance between the apiary and the pollution source, while the Cu concentrations increased linearly. The median for the elements was high: Pb—1.49 mg/kg, Cd—2.20 mg/kg, Zn—20.40 mg/kg, and Cu—3.70 mg/kg. According to Klym and Stadnytska [50], the content of heavy metals increased with the degree of the impact of industry on the environment. The levels of Zn, Cu, Pb, and Cd were the highest in honey from forested and steppe areas, lower in honey from foothills, and the lowest in mountainous areas of the Carpathian region. Dobrzański et al. [51] found that the Pb limit was exceeded in 75% of the samples of honey from apiaries located in a copper-producing region but did not observe elevated levels of Cd. The honey in the present study contained much lower levels of residues of these elements.

## 4. Conclusions

The presence of pesticide residues was detected in 90% of the analysed samples, but the concentrations did not exceed acceptable residue levels (Regulation (EC) No 396/2005 of the European Parliament and of the Council [16]). The residues of 11 substances were identified, including 5 insecticides (acetamiprid, thiacloprid, thiacloprid-amide, thiamethoxam, and dimethoate), 4 fungicides (carbendazim, azoxystrobin, tebuconazole, and boscalid) and 2 pharmacologically active substances used in veterinary medicine (DMF and total amitraz). The most frequently identified substances were thiacloprid, acetamiprid, and carbendazim. 

The analysis of heavy metals showed that the honey from apiaries located in southeastern Poland is safe for human consumption. The levels of the toxic heavy metals mercury and cadmium did not exceed the maximum level that is safe for human health. The average lead content in the varietal honey also did not exceed the accepted limit of 0.10 mg/kg. The concentrations of Zn and Cu did not deviate from those reported in the literature for specific honey varieties. 

To summarise the results of the research, the honey produced in southeastern Poland was shown to be safe for human health and consumption. The quality control of honey for the presence of chemical contaminants is crucial to the evaluation of the health safety of the product. 

## Figures and Tables

**Table 1:** List of analysed compounds.

Category of Use	Compound *
Herbicides	6-chloro-4-hydroxy-3-phenyl pyridazine (metabolite of Pyridate), 6-hydroxy bentazone (metabolite of bentazone), Acetochlor, Amidosulfuron, Asulam, Bentazone, Bifenox, Quinochlamine, Chizalofop-P-ethyl, Chizalofop-P-tefuryl, Chlomazone, Chlorosulfuron, Chlortoluron, Chloridazon, Cycloxydim, Desmedipham, Diflufenican, Dimethachlor, Etofumesate, Fenmedipham, Phenoxaprop-P-ethyl, Flazasulfuron, Florasulam, Fluazifop-P-butyl, Flufenacet, Flurochloridone, Foramsulfuron, Isoproturon, Iodosulfuron-methyl-sodium, Carbetamide, Carfentrazone-ethyl, Kletodim, Lenacyl, Linuron, Metamitron, Metazachlor, Metolachlor-S, Metribuzin, Metsulfuron-methyl, Mesosulfuron-methyl, Mesotrione, Napropamide, Nicosulfuron, Pendimethalin, Pethoxamide, Propachizafop, Propoxycarbazone sodium, Propyzamide, Prosulfocarb, Rimsulfuron, Sulfosulfuron, Sulcotrione, Tembotrione, Tepraloxydim, Terbutylazine, Thifensulfuron-methyl, Tralkoxydim
Insecticides	2,4′-DDT, 4,4′-DDD, 4,4′-DDE, 4,4′-DDT, 4,4-Methoxychlor, Acetamiprid, Aldrin, alpha-Cypermethrin, alpha-Endosulfan, alpha-HCH, azinphos-ethyl, azinphos-methyl, beta-Cyfluthrin, beta-Endosulfan, beta-HCH, Bifenthrin, Chlorantraniliprole, Chlorfenvinphos, Chlorpyriphos-methyl, Chlorpyriphos, cis-Chlordane, cis-Heptachlor epoxide, cis-Permethrin, Deltamethrin, Diazinon, Dieldrin, Diflubenzuron, Dimethoate, Endrin, Esfenvalerate, Etofenprox, Etoprophos, Fenitrothion, Fention, Fention-sulfone (metabolite of fenthion), Fention-sulfoxide (metabolite of fenthion), Fipronil, Fipronil-desulfinyl (metabolite of fipronil), Fipronil-carboxamide (metabolite of fipronil), Fipronil-sulfide (metabolite of fipronil), Fipronil-sulfone (fipronil metabolite), Flonicamid, Phoxim, Phosalon, Fosmet, HCB, Heptachlor, Heptenofos, Imidacloprid, Imidacloprid-olefin (imidacloprid metabolite), Imidacloprid-urea derivative (imidacloprid metabolite), Indoxacarb, Clothianidin, Lambda-Cyhalothrin, Lindane (gamma-HCH), Malathion, Methiocarb sulphone (methiocarb metabolite), Methiocarb sulfoxide (methiocarb metabolite), Methiocarb, Methoxyfenozide, Methidathion, MITC (Methyl isothiocyanate) (metabolite of Metam and Dazomet), Nitenpyram, Oxychlordane, Parathion ethyl, Parathion methyl, Pyrimiphos ethyl, Pyrimiphos methyl, Pyrimicarb, Pyrimicarb-desmethyl (metabolite of Pyrimicarb), Pyriproxyfen, Profenofos, Resmethrin, Endosulfan sulphate, Spinosin A, Spirodiclofen, Spirotetramat, Spirotetramat-enol (spirotetramat metabolite), Spirotetramat-enol glucoside (spirotetramat metabolite), Spirotetramat-keto hydroxy (spirotetramat metabolite) tau-Fluvalinate, Tebufenozide, Teflubenzuron, Tefluthrin, Tetramethrin, Thiacloprid, Thiacloprid-amide (metabolite of thiacloprid), Thiamethoxam, trans-Chlordane, trans-Heptachlor epoxide, trans-Permethrin, Triazinphos, zeta-Cypermethrin
Fungicides	Azoxystrobin, Bixafen, Boscalid, Bupirimate, Quinoxyfen, Chlorothalonil, Chymexazole, Cyflufenamide, Cyazofamid, Cymoxanil, Cyprodinil, Cyproconazole, Difenoconazole, Dimethomorph, Dimoxystrobin, Epoxiconazole, Fenbuconazole, Fenhexamid, Fenpropidin, Fenpropimorph, Fluchinkonazole, Fludioxonil, Flusilazole, Flutriafol, Imazalil, Ipconazole, Iprodione, Isopyrazam, Carbendazim, Carboxin, Kresoxim-methyl, Mandipropamid, Mepaniprym, Metalaxyl-M (Metalaxyl), Metconazole, Metrafenone, Myclobutanil, Pencycuron, Picoxystrobin, Pyrimethanil, Proquinazid, Prochloraz, Propamocarb, Propiconazole, Prothioconazole-desthio (a metabolite of prothioconazole), Pyraclostrobin, Pyrazophos, Silthiopham, Spiroxamine, Tebuconazole, Tetraconazole, Thiophanate-methyl, Triadimefon, Triadimenol, Trifloxystrobin, Triticonazole, Vinclozolin
Acaricides	Bifenazate, Bromopropylate, Etoxazole, Fenazaquin, Fenpyroximate, Hexithiazox, Clofentezine, Propargit, Tebufenpyrad
Veterinary drugs	Cymiazole, DMF (2,4-dimethylphenylformamide) (amitraz metabolite), DMPF (*N*-(2,4-dimethylphenyl)-*N*′-methylformamidine) (amitraz metabolite), Coumaphos
Plant growth regulators	Chlorpropham, IBA (Indolylbutyric acid), NAD (1-Naphthylacetamide)

* The limits of quantification (LOQ) of the substances are given in Supplementary Table S1.

**Table 2:** List of substances identified in honey (V—Veterinary drugs, F—Fungicides, I—Insecticides; II—Moderately hazardous, U—Unlikely to present acute hazard; LOQ—Limit of quantification (mg/kg), MRL—Maximum residue level (mg/kg)).

Compound	Category of Use	WHO Category [22]	Chemical Group	LOQ	MRL	Positive Samples	
						*n*	%
Acetamiprid	I	II	cyano-substituted neonicotinoid	0.001	0.05	26	86.66
Carbendazim	F	U	benzimidazole	0.001	1	18	60.00
Thiacloprid	I	II	cyano-substituted neonicotinoid	0.001	0.2	27	90.00
DMF (*N*-2,4-Dimethylphenyl-formamide)	V	-	-	0.005	-	17	56.66
Total amitraz	V	II	-	-	-	16	53.33
Thiacloprid-amide	I	II	cyano-substituted neonicotinoid	0.005	-	4	13.33
Thiamethoxam	I	II	nitro-substituted neonicotinoid	0.001	-	8	26.66
Dimethoate	I	II	organophosphate	0.001	-	3	10.00
Azoxystrobin	F	U	strobilurin methoxyacrylate	0.001	0.05	3	10.00
Tebuconazole	F	II	triazole	0.001	0.05	2	6.66
Boscalid	F	U	anilide pyridine-carboxamide	0.001	0.05	1	3.33

**Table 3:** Descriptive statistics of pesticide residues (mg/kg) detected in honey.

	Compoud	Mean	Median	Min	Max	25th Percentile	75th Percentile
1	Acetamiprid	0.0127	0.0065	<LOQ	0.0610	0.0020	0.0160
2	Carbendazim	0.0074	0.0020	<LOQ	0.0490	0.0000	0.0130
3	Thiacloprid	0.0527	0.0240	<LOQ	0.3370	0.0040	0.0520
4	DMF	0.0061	0.0030	<LOQ	0.0380	0.0000	0.0100
5	Total Amitraz	0.0112	0.0050	<LOQ	0.0750	0.0000	0.0160
6	Thiacloprid-Amide	0.0013	0.0000	<LOQ	0.0120	0.0000	0.0000
7	Thiamethoxam	0.0005	0.0000	<LOQ	0.0040	0.0000	0.0010
8	Dimethoate	0.0002	0.0000	<LOQ	0.0030	0.0000	0.0000
9	Azoxystrobin	0.0001	0.0000	<LOQ	0.0020	0.0000	0.0000
10	Tebuconazole	0.0001	0.0000	<LOQ	0.0020	0.0000	0.0000
11	Boscalid	0.0000	0.0000	<LOQ	0.0010	0.0000	0.0000

**Table 4:** Descriptive statistics of pesticide residues (mg/kg) detected in varietal honeys (RS—rapeseed; MF—multifloral; BW—buckwheat; HD—honeydew; LI—linden).

	Compoud	Honey	Mean	Median	Min	Max	25th Percentile	75th Percentile	Positive Samples	
									*n*	%
1	Acetamiprid	RS	0.0300	0.030	0.009	0.061	0.015	0.035	5	100.0
2	Carbendazim	RS	0.0242	0.020	0.007	0.049	0.014	0.031	5	100.0
3	Thiacloprid	RS	0.0702	0.053	0.043	0.118	0.044	0.093	5	100.0
4	DMF	RS	0.0072	0.003	<LOQ	0.027	0.002	0.004	4	80.0
5	Total Amitraz	RS	0.0144	0.006	<LOQ	0.054	0.004	0.008	4	80.0
6	Thiacloprid-Amide	RS	0.0016	0.000	<LOQ	0.008	0.000	0.000	1	20.0
7	Thiamethoxam	RS	0.0012	0.001	<LOQ	0.002	0.001	0.002	4	80.0
8	Azoxystrobin	RS	0.0004	0.000	<LOQ	0.002	0.000	0.000	1	20.0
9	Tebuconazole	RS	0.0004	0.000	<LOQ	0.002	0.000	0.000	1	20.0
10	Boscalid	RS	0.0002	0.000	<LOQ	0.001	0.000	0.000	1	20.0
	**Total**		**0.1498**							
1	Acetamiprid	MF	0.0150	0.009	0.001	0.042	0.003	0.026	10	100.0
2	Carbendazim	MF	0.0060	0.002	<LOQ	0.028	0.000	0.013	6	60.0
3	Thiacloprid	MF	0.1062	0.042	0.004	0.337	0.015	0.178	10	100.0
4	DMF	MF	0.0122	0.011	<LOQ	0.038	0.007	0.014	9	90.0
5	Total Amitraz	MF	0.0216	0.018	<LOQ	0.075	0.006	0.028	8	80.0
6	Thiacloprid-Amide	MF	0.0031	0.000	<LOQ	0.012	0.000	0.008	3	30.0
7	Thiamethoxam	MF	0.0004	0.000	<LOQ	0.002	0.000	0.000	2	20.0
8	Azoxystrobin	MF	0.0001	0.000	<LOQ	0.001	0.000	0.000	1	10.0
	**Total**		**0.1646**							
1	Acetamiprid	BW	0.0086	0.004	<LOQ	0.020	0.004	0.015	4	80.0
2	Carbendazim	BW	0.0056	0.004	<LOQ	0.018	0.000	0.006	3	60.0
3	Thiacloprid	BW	0.0122	0.005	0.002	0.027	0.003	0.024	5	100.0
4	DMF	BW	0.0012	0.000	<LOQ	0.006	0.000	0.000	1	20.0
5	Total Amitraz	BW	0.0024	0.000	<LOQ	0.012	0.000	0.000	1	20.0
6	Thiamethoxam	BW	0.0010	0.000	<LOQ	0.004	0.000	0.001	2	40.0
7	Dimethoate	BW	0.0014	0.002	<LOQ	0.003	0.000	0.002	3	60.0
	**Total**		**0.0324**							
1	Acetamiprid	HD	0.0023	0.001	<LOQ	0.007	0.000	0.005	2	50.0
2	Carbendazim	HD	0.0003	0.000	<LOQ	0.001	0.000	0.001	1	25.0
3	Thiacloprid	HD	0.0025	0.001	<LOQ	0.008	0.000	0.005	2	50.0
4	DMF	HD	0.0025	0.000	<LOQ	0.010	0.000	0.005	1	25.0
5	Total Amitraz	HD	0.0050	0.000	<LOQ	0.020	0.000	0.010	1	25.0
	**Total**		**0.0125**							
1	Acetamiprid	LI	0.0047	0.002	<LOQ	0.016	0.001	0.007	5	83.3
2	Carbendazim	LI	0.0018	0.002	<LOQ	0.005	0.000	0.003	3	50.0
3	Thiacloprid	LI	0.0160	0.015	<LOQ	0.032	0.003	0.031	5	83.3
4	DMF	LI	0.0013	0.000	<LOQ	0.005	0.000	0.003	2	33.3
5	Total Amitraz	LI	0.0027	0.000	<LOQ	0.010	0.000	0.006	2	33.3
6	Azoxystrobin	LI	0.0002	0.000	<LOQ	0.001	0.000	0.000	1	16.6
7	Tebuconazole	LI	0.0002	0.000	<LOQ	0.001	0.000	0.000	1	16.6
	**Total**		**0.0268**							

## Data Availability

Data will be made available upon reasonable request by the corresponding author.

## Supplementary Materials

The following supporting information can be downloaded at: https://www.mdpi.com/article/10.3390/foods11152362/s1, Table S1: The limits of quantification (LOQ) of the pesticides.
