# Supplementary material for: Residues of Pesticides and Heavy Metals in Polish Varietal Honey
Source: Foods. 2022 Aug 6;11(15):2362. doi: 10.3390/foods11152362 (PMC9368611; doi:10.3390/foods11152362)
Supplement: Supplementary file 1 [file foods-11-02362-s001.zip › foods-1825426-supplementary.pdf]

Table S1. Limit of quantification (LOQ) for the analysed pesticides.

Compound	LOQ (mg/kg)
2,4'-DDT; 4,4'-DDD; 4,4'-DDE; 4,4'-DDT; Acetamiprid; Aldrin; alpha-HCH; Amidosulfuron; Asulam; Azoxystrobin; beta-HCH; Bifenthrin; Bixafen; Boscalid; Bupirimate; Chlomazone; Chlorantraniliprole; Chlorpyrifos-methyl; Chlorpropham; Chlorosulfuron; Chlortoluron; Chloridazon; cis-Chlordane; cis-Epoxide heptachlor; Cymoxanil; Cyproconazole; Desmedipham; Difenoconazole; Dimethachlor; Dimethoate; Dimethomorph; Dimoxystrobin; DMF (2,4-dimethylphenylformamide) (amitraz metabolite); Etofenprox; Fenmedipham; Fenpropimorph; Fention-sulfoxide (fenthion metabolite); Fipronil; Fipronil-desulfinyl (fipronil metabolite); Flazasulfuron; Florasulam; Fluchinkonazole; Flufenacet; Flusilazole; Foramsulfuron; HCB; Heptachlor; Heptenofos; Imidacloprid; Imidacloprid-urea derivative (metabolite of imidacloprid); Carbendazim; Clothianidin; Coumaphos; Mandipropamid; Metalaxyl-M (Metalaxyl); Metazachlor; Methiocarb; Methiocarb sulfone (methiocarb metabolite); Methiocarb sulfoxide (methiocarb metabolite); Metconazole; Metolachlor-S; Metrafenone; Metsulfuron-methyl; Methidation; Mesosulfuron-methyl; NAD (1-Naphthylacetamide); Napropamide; Nicosulfuron; Oxychlordane; Parathion-methyl; Parathion-methyl; Pencycuron; Pethoxamide; Pyraclostrobin; Pirimicarb; Pirimicarb-desmethyl (metabolite of pirimicarb); Prochloraz; Propiconazole; Propyzamide; Rimsulfuron; Silthiopham; Spirotetramat; Spirotetramat-enol (metabolite of spirotetramat); Sulfosulfuron; tau-Fluvalinate; Tebuconazole; Tefluthrin; Terbuthylazine; Tetraconazole; Thiamethoxam; Thiacloprid; Thifensulfuron-methyl; trans-Chlordane; Triadimefon; Vinclozolin	0.001
Acetochlora; alpha-Cypermethrin; alpha-Endosulfan; azinphos-ethyl; azinphos-methyl; 6-hydroxy bentazone (bentazone metabolite); beta-Cyfluthrin; beta-Endosulfan; Bifenazate; Bifenox; Bromopropylate; Quinochlamine; Chizalofop-P-ethyl; Chlorfenvinphos; Chlorpyrifos; cis-Permethrin; Cyflufenamid; lambda-Cyhalothrin; Cyazofamid; Cycloxydim; Cyprodinil; Deltamethrin; Diazinon, Dieldrin; Diflufenican; Endrin; Epoxiconazole; Esfenvalerate; Etoprophos; Fenazaquin; Fenbuconazole; Fenhexamid; Fenitrothion, Fenpropidin; Fention; Fention-sulfide (a metabolite of fenthion); Fipronil-sulfide (metabolite of fipronil); Flonicamid; Fludioxonil; Flurochloridone; Flutriafol; Fosalon; Fosmet, Hexithiazox; IBA (Indolylbutyric acid); Imazalil; Imidacloprid-olefin (metabolite of imidacloprid); Ipconazole; Isoproturon; Isopyrazam; Iodosulfuron-methyl sodium; Carbetamide; Carboxin; Ethyl carfentrazone; Clethodim; Clofentezine; Methyl msime; Lenacyl; Lindane (gamma-HCH); Linuron; Malathion; Mepaniprym; Metamitron; Methoxyfenozide; Metribuzin; MITC (methyl isothiocyanate) (metabolite of metam and dazomet); Myclobutanil; Pendimethalin; Profenofos; Pyrazophos; Pyrimethanil; Pyrimiphos ethyl; Pyrimiphos-methyl; Prothioconazole-desthio (metabolite of prothioconazole); Endosulfan sulfate; Spinosin A; Spirodiclofen; Spirotetramat-enol glucoside (metabolite of spirotetramat); Spirotetramat-keto hydroxy (metabolite of spirotetramat); Tepraloxydim; Tetramethrin; Thiacloprid-amide (metabolite of thiacloprid); Tralkoxydim; trans-Epoxide of heptachlor; trans-Permethrin; Trifloxystrobin; Triticonazole; zeta-Cypermethrin	0.005
4,4-Methoxychlor; 6-chloro-4-hydroxy-3-phenyl pyridazine (metabolite of Pyridate); Chlorothalonil; Cymiazole; Diflubenzuron; Ethofumesate; Etoxazole; Phenoxaprop-P-ethyl; Fipronil-sulfone (metabolite of fipronil); Fipronil-carboxamide (metabolite of fipronil); Fluazifop-P-butyl; Phoxim; Indoxacarb; Iprodione; Mesotrione; Nitenpyram; Picoxystrobin; Proquinazid; Propachizafop; Propamocarb; Prosulfocarb; Resmethrin; Spiroxamine; Tebufenozide; Tebufenpyrad; Triadimenol; Triazinphos	0.01

Bentazone; Quinoxyfen; Chizalofop-P-tefuryl; DMPF (N-(2,4-dimethylphenyl)-N'-methylformamidine) (metabolite of amitraz); Fenpyroximate; Chymexazol; Pyriproxyfen; Propargit; Teflubenzuron; Tembotrione; Thiophanate-methyl	0.05
Sodium propoxycarbazone; Sulcotrione	0.1
